# Experiments in Globalisation, Food Security and Land Use Decision Making

**DOI:** 10.1371/journal.pone.0114213

**Published:** 2014-12-01

**Authors:** Calum Brown, Dave Murray-Rust, Jasper van Vliet, Shah Jamal Alam, Peter H. Verburg, Mark D. Rounsevell

**Affiliations:** 1 School of GeoSciences, University of Edinburgh, Edinburgh, EH8 9XP, United Kingdom; 2 Institute for Environmental Studies, VU University Amsterdam, Amsterdam, The Netherlands; University of Warwick, United Kingdom

## Abstract

The globalisation of trade affects land use, food production and environments around the world. In principle, globalisation can maximise productivity and efficiency if competition prompts specialisation on the basis of productive capacity. In reality, however, such specialisation is often constrained by practical or political barriers, including those intended to ensure national or regional food security. These are likely to produce globally sub-optimal distributions of land uses. Both outcomes are subject to the responses of individual land managers to economic and environmental stimuli, and these responses are known to be variable and often (economically) irrational. We investigate the consequences of stylised food security policies and globalisation of agricultural markets on land use patterns under a variety of modelled forms of land manager behaviour, including variation in production levels, tenacity, land use intensity and multi-functionality. We find that a system entirely dedicated to regional food security is inferior to an entirely globalised system in terms of overall production levels, but that several forms of behaviour limit the difference between the two, and that variations in land use intensity and functionality can substantially increase the provision of food and other ecosystem services in both cases. We also find emergent behaviour that results in the abandonment of productive land, the slowing of rates of land use change and the fragmentation or, conversely, concentration of land uses following changes in demand levels.

## Introduction

In neoclassical economic theory, globalisation underpinned by free trade will produce an optimal distribution of land uses, so that goods and services are produced wherever it is most efficient – and cheapest – to do so, providing benefits throughout the supply chain [1],[2]. This implies separation of sites of production and consumption of goods and services, with major consequences for existing patterns of agricultural land uses in particular [3]. While some governments and international bodies promote trade of this kind (e.g. [4]–[6]), it is more commonly opposed in the interests of national or regional food security, to balance the interests of productive and economically important industries, conserve biodiversity, or respect public demand for various land uses [7]–[9].

Policies that aim to ensure food security notably include the European Union's Common Agricultural Policy (CAP). Intended to maintain a level of self-sufficiency in Europe, the CAP provides support for European agriculture at the expense of potentially more efficient production elsewhere in the world [10]. If production levels and land use efficiency are maximised by global free trade amongst purely rational agents (who are in full possession of relevant knowledge and account for environmental externalities), directed interventions of this kind lead only to sub-optimal outcomes by reducing and slowing the global influence on local land use change. Resulting land use configurations are, in theory, less efficient, productive and profitable than those arising from unfettered trade in agricultural goods. In the case of the CAP, regional overproduction of food and environmentally damaging intensification of agriculture has been stimulated at times, alongside land abandonment in marginal areas (e.g. [11],[12]).

The choice between maximised global food production that risks fundamentally altering regional land systems and regional food security that risks reducing overall production levels and land use efficiency is obviously not clear cut. Not only do externalities such as social or environmental effects complicate the identification of an optimal or ‘best’ land use strategy [13],[14], but practical constraints on the production and supply of goods and services make perfectly globalised systems impossible to establish [3],[9]. Furthermore, human responses can entirely change a strategy's outcome. The beliefs, experience and behaviour of individual land managers are known to be strong determinants of land use change, and these interact with political interventions in complex ways [10],[15]. For example, many studies have found that farmers' individual characteristics affect the (heavily-legislated) process of agricultural land use change, identifying numerous personal or cultural factors that can have a decisive effect on land use decisions (e.g. [16]). These effects include land managers resisting policies that are not consistent with their own beliefs or desires (e.g. [17]). The speed and extent of uptake of particular schemes has been found to vary dramatically as a result [18],[19]. Individual preferences can also produce emergent societal influences such as support for local food or recreation (e.g. [20]), or, indeed, opposition to globalisation expressed through democratic processes (e.g. [21]).

Behavioural effects are likely to be especially strong in a changing system. For example, land managers who differ in their ability or willingness to meet demands for particular services are likely to show strongly divergent responses to changing demand levels [22], while those who are most dependent upon natural resources need to be most adaptable to climate change [23]. In theory, globalised systems are adept at coping with such changes in demand or contextual factors, allowing compensatory adjustments to spread quickly following a disturbance somewhere in the system [24]. However, the behaviour of individual land managers has the potential to undermine this process, and so the true implications of global and regional approaches to food security under climatic and societal change remain uncertain.

Despite the importance of these issues, the effects of land managers' responses to change in globalised and regionalised land systems have not been fully investigated beyond analysis with macro-level models based only on economic theory [2]. Methods do exist to investigate these effects, and foremost among these are Agent-Based Models (ABMs) that attempt to describe the effects of individual behaviours on complex systems [25]–[28]. Nevertheless, to our knowledge, ABMs have not been used to investigate, systematically, responses to policies dedicated to maximising global food production or ensuring regional food security in dynamic land use systems.

Here we use a set of simulation experiments with a land use ABM to examine the effects of land managers' individual behaviours on the configurations, productivities, and efficiencies of land uses in idealised global and regional land use systems (in which globalisation and regionalisation occur perfectly, with either completely free or completely limited trade in goods and services). Specifically, we investigate the role of land manager behaviour that is not strictly ‘rational’ in driving deviations from optimal land use configurations (i.e., configurations where production per unit area is maximised), the potential consequences of this for food security in globalised and regionalised systems, and the effects of multi-functional land use on the production of food and other ecosystem services.

## Methods

### 1. Overview of model

The ‘Competition for Resources between Agent Functional Types’ (CRAFTY) model framework used in this study is based on the demand and supply of ecosystem services (ES) that are produced by agents representing land managers. Demand levels are introduced exogenously to represent societal desires and requirements, and agents compete to satisfy these on the basis of their productive ability and behavioural characteristics. Agents utilise locational capitals that describe the productive potential of land in order to produce ES according to defined production functions (see below). ES can represent tangible goods such as food and timber or broader services such as recreation, cultural landscapes and aesthetic value (for a full description of CRAFTY see [29]).

Agents are characterised according to the Agent Functional Type concept [26],[30], which suggests that land managers may be grouped by behaviour or by their productive response to environmental (locational) conditions, in analogy to plant functional types (PFTs) in ecosystem models. Each AFT has a different production function, describing its ability to utilise particular capitals in order to produce particular ES, and may also have additional distinct behavioural settings. Within each type, agents may be homogeneous or heterogeneous.

A fundamental basis for agent behaviour in the model is provided by *abandonment* and *competition* thresholds that describe agents' willingness to abandon or relinquish land. Agents in the model compete for available land parcels (represented by cells in a grid) based on these two parameters. The inspiration for this simple behavioural representation comes from several studies that have suggested that a wide range of behaviours are reducible to a small number of dimensions of this kind (e.g. [16],[31]). We use these thresholds to represent real-world variation in personal characteristics or decision-making strategies that alter land managers' dedication to their land use, and they can be used in this way to encapsulate variation in culture, profit-sensitivity, available labour pool, personal financial resources and other similar factors as appropriate. They can also be used to account for costs of production or change of land use, when a minimum return is required to avoid a net loss being made. Also included are parameters that control an agent's ability to search for suitable cells and those describing an agent's production function.

Once parameterised, the model runs through a series of ‘timesteps’, each of which typically represents a single year. At each timestep, searches are undertaken by a typical agent of each type, in order to identify cells where their productive ability is maximised. Both the number of searches carried out and the number of cells considered during each search are specified (Table 1). Searched cells are ranked after each search according to the competitiveness of that AFT at that location, and individual agents then attempt to take over these cells, in order, until one cell is taken over or the list of cells is exhausted. Competitiveness is calculated on the basis of an AFT's mean (or uniform) ES production, which is given a utility value via a function linking unmet demand and production levels.

**Table 1:** Descriptions of the main parameters in the model.

PARAMETER	INTERPRETATION
Capital sensitivity	Quantification of agent's dependence on a capital for the production of a service
Productive ability	Proportion of a productive unit attained by agent under ‘perfect’ capital conditions
Search iterations	Number of separate search events carried out by each agent type
Cells per search	Number of cells considered at each search iteration
Abandonment threshold	Minimum utility value an agent will accept before abandoning land
Competition threshold	Maximum competitive disadvantage (in terms of utility difference) an agent will tolerate before relinquishing land to a competitor

Parameter names and interpretations are shown here, with values for each experiment given in Table 3.

Agents continue production as long as their utility value is greater than their abandonment threshold (the value representing the lower limit at which an agent can or will persist with a land use), and will only relinquish land to a competitor with a utility that exceeds their own by more than their competition threshold. Agents therefore succeed in taking over a cell when that cell is currently unmanaged (including when the previous cell occupant has just abandoned the cell) or when they are able to outcompete the current occupant by some margin, at which point their own land use is assumed to be immediately implemented. In this way, agents can be parameterised as non-behavioural land use optimisers, or alternatively, as active intermediaries in the demand/supply chain.

### 2. Experimental setup

**Figure 1:**
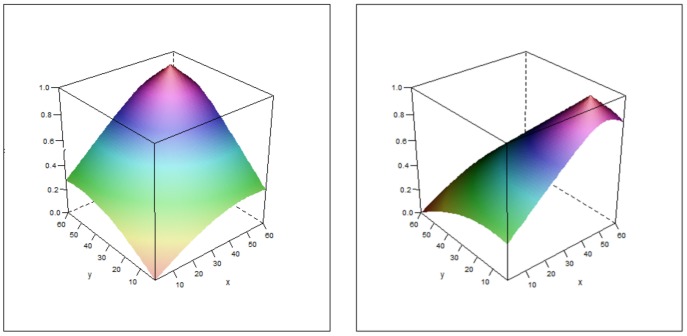
Variation in productivity capitals across the modelled arena. Crop productivity is shown on the left and natural capital on the right. Both are maximised on the right-hand-side of the arena in order to allow separation of agent types while generating competition for the most productive cells.

Our experiments are designed to investigate effects of land manager behaviour on land use under globalisation and regionalisation of demand for food and other ecosystem services. We start with a simple baseline model intended to investigate the effects of regionalisation and changing demand levels in the absence of any confounding processes. We then add complexity to this model as detailed below. Throughout, we use the same modelled world (or arena), represented by a 60 by 60 cell grid, with two distinct capitals (crop productivity and natural capital) that vary across the grid. Under regionalisation, this grid is divided into four 30 by 30 cell regions. The maximum values of both capitals are located on the same side of the arena (Fig. 1) to generate competition between agents for highly productive areas.

Each cell in the world may be managed by a single agent, and agents are distributed across the world randomly at the start of each simulation. The agents then compete for land over the course of 25 timesteps. We run 30 realisations of each experimental setup in order to construct envelopes of results that provide information about the relative strength of stochastic and systematic variation within and between simulations. We also monitor the time taken for productivities to converge to a steady state across realisations of each experiment, both from the initial agent distribution and following a change in demand levels (with a steady state defined as the state where the annual variation in ES supply between realisations is greater than the difference between annual mean ES supplies across realisations). The rationale and parameter settings for each simulation are given in Tables 2 and 3.

**Table 2:** Descriptions and rationales for the experiments.

EXPERIMENT(S)	DESCRIPTION	RATIONALE
1	Baseline experiment	To establish land use configurations in the absence of any behaviour.
2–5	Variations in abandonment thresholds	To investigate effects of raised abandonment thresholds (unwillingness to accept low returns) in either (2–3) or both (4) agent types, and when individual variation occurs (5).
6	Variation in competition thresholds	To investigate effects of raised competition thresholds (unwillingness to relinquish land), with individual variation.
7	Reduced ability to search for cells	To establish effects of a reduction in agents' ability to search for cells on which to compete.
8	Decreased sensitivity to demand levels (exponential form of utility functions to give positive utility in the case of over-supply of services)	To investigate effects of (a) insensitivity to demand levels, or (b) personal motivation for production, or (c) a cross-regional market giving value to overproduction.
9–13	Variable intensities of land use with and without additional behaviour as above	To investigate how above effects change when different intensities of land uses are available.
14–19	Multifunctional and variable intensity land uses with and without additional behaviour as above.	To investigate how land use multi-functionality changes the above effects under different behaviours.

All experiments are performed under both static and dynamic demand. Parameter settings are given in Table 3.

**Table 3:** Parameter settings used in the experiments.

EXPERIMENT	HIF	MIF	LIF	CONS	SEARCH	CELLS/SEARCH	UTILITY FUNCTION
	AT; CT	AT; CT	AT; CT	AT; CT	ITS.		
1	0.0; 0.0	NA	NA	0.0; 0.0	5000	10	*y = 3x*
2	**0.2**; 0.0	NA	NA	0.0; 0.0	5000	10	*y = 3x*
3	0.0; 0.0	NA	NA	**0.2**; 0.0	5000	10	*y = 3x*
4	**0.2**; 0.0	NA	NA	**0.2**; 0.0	5000	10	*y = 3x*
5	0.0; 0.0	NA	NA	**N(0.2,0.03)**; 0.0	5000	10	*y = 3x*
6	0.0; 0.0	NA	NA	0.0; **N(0.2,0.03)**	5000	10	*y = 3x*
7	0.0; 0.0	NA	NA	0.0; 0.0	**100**	10	*y = 3x*
8	0.0; 0.0	NA	NA	0.0; 0.0	5000	10	***y = e^x^***
9	0.0; 0.0	**0.0; 0.0**	**0.0; 0.0**	0.0; 0.0	5000	10	*y = 3x*
10	**0.2**; 0.0	**0.0; 0.0**	**0.0; 0.0**	**0.2**; 0.0	5000	10	*y = 3x*
11	0.0; 0.0	**0.0; 0.1**	**0.0; 0.2**	0.0; 0.0	5000	10	*y = 3x*
12	0.0; 0.0	**0.0; 0.0**	**0.0; 0.0**	0.0; 0.0	5000	10	***y = e^x^***
13	0.0; 0.0	**0.0; 0.1**	**0.0; 0.2**	0.0; 0.0	5000	10	***y = e^x^***
14	0.0; 0.0	**0.0; 0.0 (Multi)**	**0.0; 0.0 (Multi)**	0.0; 0.0	5000	10	*y = 3x*
15	**0.2**; 0.0	**0.0; 0.0 (Multi)**	**0.0; 0.0 (Multi)**	**0.2**; 0.0	5000	10	*y = 3x*
16	**N(0.2,0.03)**; 0.0	**0.0; 0.0 (Multi)**	**0.0; 0.0 (Multi)**	**N(0.2,0.03)**; 0.0	5000	10	*y = 3x*
17	0.0; 0.0	**N(0.2,0.03); 0.0 (Multi)**	**N(0.2,0.03); 0.0 (Multi)**	0.0; 0.0	5000	10	*y = 3x*
18	0.0; 0.0	**0.0; 0.1; Multi**	**0.0; 0.2; Multi**	0.0; 0.0	5000	10	*y = 3x*
19	0.0; 0.0	**0.0; 0.0 (Multi)**	**0.0; 0.0; (Multi)**	0.0; 0.0	5000	10	***y = e^x^***

Settings that are altered relative to Experiment 1 in each case are in bold. Each Experiment is run four times (labelled as *a*, *b*, c, *d*); once for each combination of static and dynamic demand with globalised and regionalised configurations (a =  static globalised, b =  dynamic globalised, c =  static regionalised, d =  dynamic regionalised). N(*y,z*) denotes a Gaussian distribution with mean *y* and standard deviation *z*. Agent types are denoted as follows: HIF  =  high-intensity farmers; MIF  =  mid-intensity farmers; LIF  =  low-intensity farmers; Cons  =  conservationists. AT and CT refer to Abandonment Thresholds and Competition Thresholds, respectively.

### 3. Simulation schedule

Our baseline simulation is one in which two agent functional types (AFTs) – ‘farmers’ and ‘conservationists’ – compete to satisfy abstract demands for food and recreation. The identities of these AFTs are arbitrary and are used to differentiate the two rather than to link them to real-world characteristics of such land managers. Farmer productivity depends on the crop productivity capital and conservationist productivity depends on the natural amenity capital. Both AFTs are capable of producing a single ‘unit’ of their ES under optimum conditions (where the relevant capital is maximised, at a value of 1) (Table 4).

**Table 4:** Capital sensitivities and production levels for each agent type used in the experiments.

AGENT TYPE	SENSITIVITY TO CROP PRODUCTIVITY	SENSITIVITY TO NATURAL CAPITAL	FOOD PRODUCTION	RECREATION PRODUCTION
High Intensity Farmer	1.0	0.0	1.0	0.0
Mid Intensity Farmer (1)	0.5	0.0	0.5	0.0
Low Intensity Farmer (1)	0.25	0.0	0.25	0.0
Mid Intensity Farmer (2)	0.75	0.20	0.75	0.15
Low Intensity Farmer (2)	0.35	0.4	0.35	0.4
Conservationist	0.0	1.0	0.0	1.0

Mid and low intensity farmers (2) were multifunctional, while (1) were not.

Initially, utility for both services is represented by a linear function *y = ax*, where *y* is the utility for the production of a unit of an ES, and *x* is the unmet demand for this ES. A linear function is chosen for its generality and interpretability, with increasing levels of unmet demand generating steady increases in utility values. Negative values are set to zero, so that overproduction of an ES is neither to the benefit nor detriment of an agent (the value of the gradient *a* in this linear relationship is arbitrary and set here to 3.0 for both services; changes in this value would alter the rate at which responses to changes in demand levels occurred, but not the relative competitiveness of modelled service production). Abandonment and competition thresholds are initially set to 0.0, so that agents relinquish land when they do not have a positive competitiveness or when another agent has a higher competitiveness. At each timestep, each AFT undertakes 5,000 search iterations of 10 randomly-selected cells and then attempts to take over these cells.

Demand levels are set so that an optimum agent configuration is almost capable of satisfying global demands for food and recreation, which are equal and static (so that every cell is required for production and is subject to competition between agents). In order to investigate the effects of dynamic demand we then introduce a step-change in demand during the relevant simulations, with demand for recreation dropping by 75% after 11 timesteps. Subsequently, these same static and dynamic demands are divided between four equally-sized regions in order to investigate the effects of regionalisation caused by policies dedicated to regional food security. Beyond this, we include no political or economic barriers to the establishment of optimal land use patterns (policies that slow or prevent large-scale land use change are not simulated, for instance), so that the effects of modelled behaviour can be isolated.

### 4. Behavioural variations

Using the above basic settings, we vary model parameters to introduce individual and typological agent behaviour, and to relax the distinction between regionalised and globalised systems. We first vary abandonment thresholds, systematically and stochastically, for both AFTs. We then similarly vary competition thresholds, before altering agents' abilities to search and compete for cells, and changing the form of the utility functions. The parameter values used in each case and rationales for these changes are given in Tables 2 and 3.

Following these behavioural variations, we introduce two additional AFTs – mid- and low-intensity farmers - to the simulations. At first, these types produce only food, and are distinguished from high-intensity farmers by their reduced sensitivity to capital levels and their reduced productive ability. Later, we allow these agents to adopt multi-functional land uses, so that, in addition to food, they also produce recreation while having limited sensitivity to the relevant capitals (Table 4). In both cases, we introduce some of the above behavioural variations, which we do not attempt to link to particular human behaviours or characteristics, because the nature, number and complexity of these factors effectively preclude the identification of such links (e.g. [26],[32]). Instead, we use systematic and stochastic variation between and within AFTs to identify the general effect of broad behavioural variations; an approach thought to be suitable for a complex system of this kind [16],[33]. We discuss links between our simulated variations and real-world land manager behaviour in Tables 2 and 5, and in the Discussion section.

**Table 5:** Summary of the dominant effects of each form of behavioural variation investigated in the experiments (see Table 2 for further information on behavioural variations).

BEHAVIOUR	PARAMETERISATION (EXPERIMENTS)	DOMINANT EFFECT UNDER STATIC DEMAND	DOMINANT EFFECT UNDER DYNAMIC DEMAND
Unwillingness to persist with land uses that offer low returns (e.g. lack of dedication/reliance on particular land use; motivated by economic concerns; innovative).	Raised abandonment threshold (2–5)	Reduced production levels, abandonment of relatively productive land under regionalisation especially with individual variation.	Increases productive efficiency as agents with higher thresholds retreat to most productive land
Unwillingness to relinquish land to more competitive agent (e.g. dedication to land use through sense of personal or cultural responsibility).	Raised competition threshold (6)	No clear effect beyond mixing of agents with different thresholds	No clear effect
Limited ability to search for cells on which to compete (e.g. imperfect knowledge of the ‘world’).	Lower number of searches permitted per time step (7)	Delays establishment of stable land use configuration	Agents more widely dispersed following demand level changes
Limited sensitivity to demand levels (e.g. production for personal reasons or over long time-scales; some trade of surpluses between regions).	Exponential demand functions (8)	Overall production levels increased and most productive land in use	Similar but weaker effect as under static demand
Ability to vary land use intensity (e.g. responses in inputs or labour to changing market conditions).	Extra agent types with differing land use intensities (9)	Cyclical competition for land	Cyclical competition for land
Ability to vary land use intensity and other behaviours	Extra agent types and parameterisations similar to Experiments 2–9 (10–13)	Lower intensities favoured by some behaviours; production levels decline. Exponential utilities drive low intensity agents out	As static, but more land under management
Ability to produce multiple services (e.g. decision to produce non-essential services or exploit full potential of land).	Extra, multifunctional agent types (14)	Increased (cyclical) competition, but higher overall production under regionalisation	Multifunctional agents drive out producers of single service for which demand is low
Ability to produce multiple services and other behaviours	Extra, multifunctional agent types and parameterisations similar to Experiments 2–9 (15–19)	Competition and production levels smoothed, smaller difference between globalised and regionalised cases. Multifunctional agents with high competition thresholds dominated and improved regional supply.	Abandoned land found in least productive areas under globalisation but in most productive areas under regionalisation. Exponential utilities maximise supply of services.

## Results

**Figure 2:**
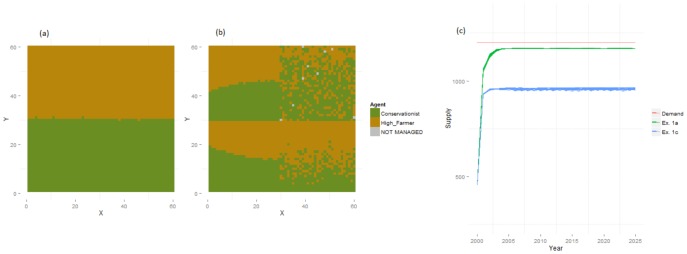
Baseline land use and supply levels results (Experiment 1) under constant levels of demand for services. Land use maps are shown for for Experiments 1a (a) and 1c (b) along with the corresponding supply of food produced in each (c).

A full description of the results is given here, while principal findings are summarised in the Discussion section below, and in Table 5. The basic model setup (Experiment 1) was used to explore the effects of globalised and regionalised demand in the absence of any behaviour, providing a baseline for further experiments. Agents in the static globalised system quickly converged to a near-optimal configuration (Table S1 in file S1) in which each type occupied areas where it was particularly productive (Fig. 2a; Table 4). This allowed supply levels for food and recreation to remain stable and equal, nearly meeting global demands. Regionalisation of this system produced faster convergence but a less clear division between AFTs (Fig. 2b), as agents attempted to meet demands within each region and so utilised land that was less productive for their specific ES. In regions with low capital levels, areas occupied by each AFT remained distinct because supply could not match demand and, consequently, large differences in competitiveness occurred. In the most productive regions, however, demand could easily be satisfied and there was no inducement for agents to seek out the most productive cells. Some locations were abandoned as a result, and global supply (or production) was found to decline sharply (Fig. 2c).

**Figure 3:**
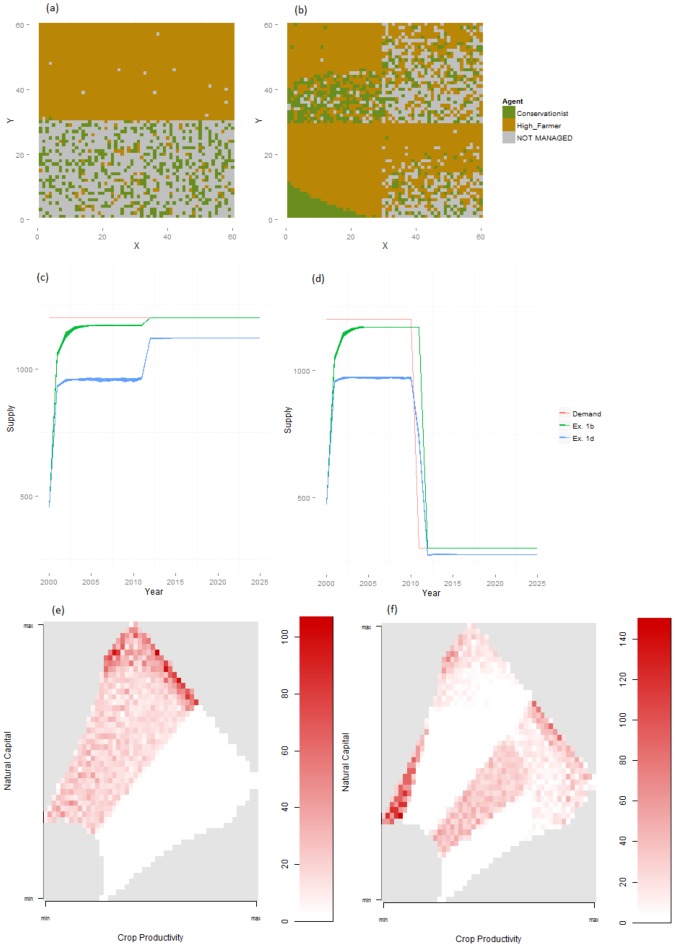
Baseline results (Experiment 1) following drop in demand for recreation. Final land use maps are shown for Experiments 1b (a) and 1d (b), following a drop in demand for recreation. The corresponding levels of demand and supply of food and recreation services are shown in (c) and (d) respectively. The distribution of conservationist agents in capital space is shown for Experiment 1b in (e) and for Experiment 1d in (f). Uniform grey areas of capital space in (e) and (f) do not occur in the modelled arena.

When demand for recreation was suddenly reduced, both the globalised and regionalised systems adapted quickly (Table S1 in File S1). Conservationist agents abandoned a large number of cells (particularly in productive regions, under regionalisation, as only a few cells were needed to meet the reduced demand), and farmer agents took some of these cells over. Following this, as the supply of food approached demand levels and utility values for farmers declined, a smaller number of more productive cells, where farmers were still more likely to occur, were abandoned (increasing costs of land conversion would slow and, if large enough, prevent this by discouraging the adoption of more marginal cells) (Figs. 3a & b). Production of both services met or almost met demands in globalised and regionalised systems (Figs. 3c & d), though conservationists remained in areas of high natural amenity capital in the global case but only in areas of low natural amenity capital in the regional case (Figs. 3e & f). This was due to the differences in initial configurations and because individual agents had no reason to prefer production in a few productive cells over production in many marginal cells, as long as their thresholds for competition and abandonment were satisfied. Consequently land was predominantly abandoned in areas that were highly productive for both services in the regional case.

Experiments 2–7 all showed a decline in total production levels and the establishment of sub-optimal land use configurations under different forms of modelled behaviour (some did, however, show faster convergence times following demand level changes). These experiments also revealed some specific and unexpected responses to regionalisation and dynamic demand levels (Table 5).

**Figure 4:**
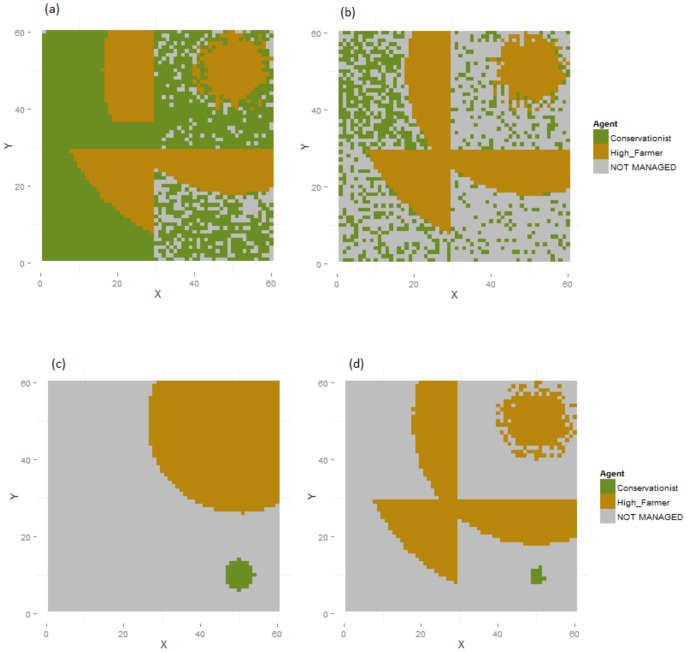
Effects of variation in abandonment thresholds (Experiments 2 & 3) on response to drop in demand for recreation. Final land use maps are shown for Experiments 2c (a), 2d (b), 3b (c) and 3d (d), showing the responses of conservationists to a drop in demand for recreation under different abandonment thresholds. Farmer agents have higher abandonment thresholds in Experiment 2 and conservationists in Experiment 3, respectively producing dispersed and concentrated patterns of conservationist land use.

Increased typological abandonment thresholds (modelling an unwillingness to persist with low-utility land management; Experiments 2–5) restricted the land used by AFTs with higher thresholds, and consequently reduced total production levels (Figure 4). When demand levels for the AFT with the lowest threshold (in this case conservationists) dropped in a globalised world, that type abandoned land (in a single time-step) in an apparently random pattern. Under regionalisation, the majority of abandoned land was highly productive (with high levels of natural amenity capital) (Fig. 4b). When demand levels for the AFT with the highest threshold (farmers) dropped, in contrast, agents of that type persisted primarily in the most productive cells (which they were already concentrated around), entirely abandoning every other region (Figs. 4c & 4d). This pattern of abandonment was reinforced by the tendency of high abandonment thresholds to discourage adoption of marginal cells, and did not occur under random individual variation in abandonment or competition thresholds (Experiments 5 & 6; Fig. S1 in File S1).

**Figure 5:**
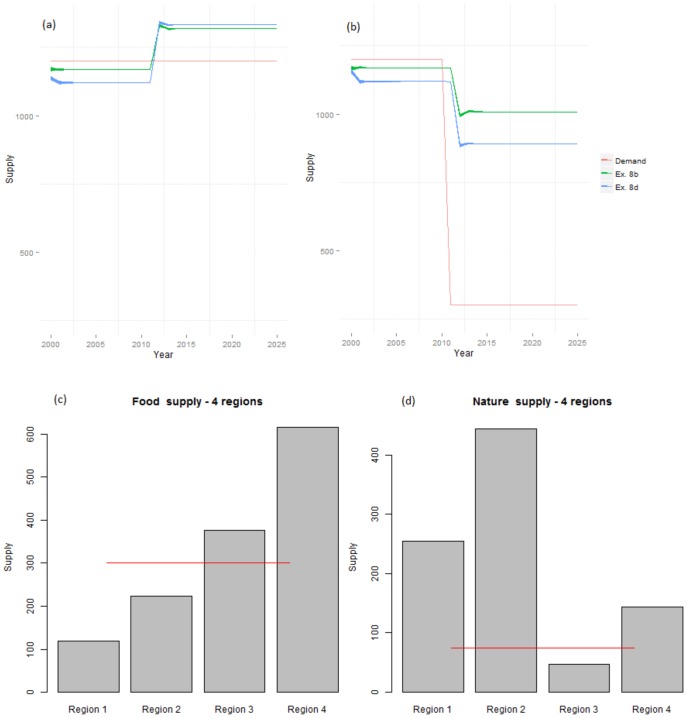
Global and regional supply levels under decreased sensitivity to demand levels (Experiment 8). Global supply of food (a) and recreation (b) under dynamic recreation demand levels in Experiments 8b and 8d, and regional supply of food (c) and recreation (d) in Experiment 8d. Decreased sensitivity to demand levels is modelled through exponential utility functions, and resulted in overproduction in the most productive regions. Red lines are demand levels, which are shown following the drop in recreation demand in (c) and (d).

The effect of limiting the search ability of agents (Experiment 7) was to delay the initial establishment of stable land use configurations (this is apparent visually, although our measure of convergence did not detect this due to large inter-simulation variability and the slow rate of change), and to produce less concentrated and distinct final agent distributions (Fig. S2 in File S1). When exponential utility functions were used to model the effects of agent insensitivity to demand levels or limited trade of regional surpluses (Experiment 8), the supply of ES was found to increase in all simulations, especially in the regional cases (Figs. 5a & 5b), where agent locations were influenced by productivity as well as regional demand. Productive regions therefore over-produced both ES (Figs. 5c & 5d), and no land was abandoned in any region.

**Figure 6:**
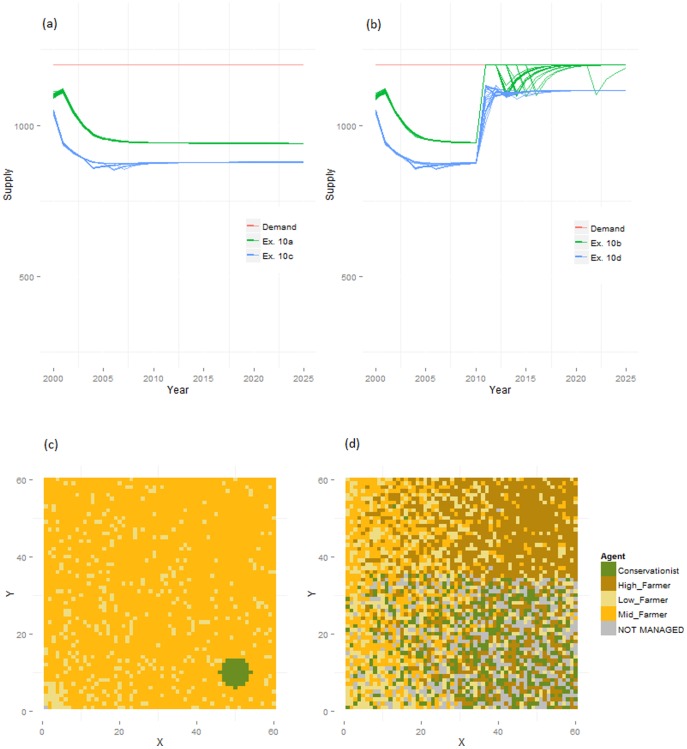
Supply levels and land use maps following the introduction of multifunctional agents. Supply of food in Experiment 10 under static demand (a) and dynamic demand for recreation (b), and final land use maps under global dynamic demand in Experiments 10b (c) and 11b (d), showing the difference in the response of conservationists to the drop in demand for recreation as their abandonment thresholds are varied.

The remaining simulations addressed the effects of variations in intensity and multi-functionality of land uses by adding additional AFTs to the simulated world. Experiment 9 established baseline results for the inclusion of mid- and low-intensity farmers (Table 4). In this experiment, both new AFTs were quickly eliminated from the global simulations following cyclical competition for marginal land, but mid-intensity farmers retained the least productive land in the regional simulations. Raising the abandonment thresholds of the original high-intensity AFTs (Experiment 10) and the competition thresholds of the new, lower intensity AFTs (Experiment 11) allowed mid- and low-intensity farmers to manage a larger number of cells. This resulted in lower food production under static demand, but reduced abandonment of marginal land under dynamic demand because agents adapted land use intensity to local conditions (Fig. 6) (this effect was less marked under exponential utility functions, Experiments 12 and 13).

We next added multi-functionality to the simulations by allowing mid- and low-intensity farmers to supply recreation as well as food (but at lower total productivities than high-intensity agents; Table 4). In the baseline simulation (Experiment 14), this resulted in low intensity producers broadly specialising in areas of low productivity and, under dynamic demand, outcompeting conservationists (Figs. S3a & S3b in File S1). Although agent locations and supply levels were not stable, the supply of both services under regionalisation was improved (Figs. S3c & S3d in File S1).

**Figure 7:**
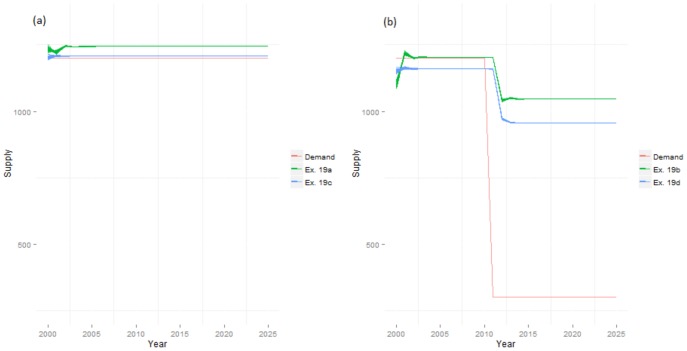
Demand and supply levels with agent multifunctionality and reduced sensitivity to demand (Experiment 19). Food supply under static demand in Experiments 19a and 19c (a) and nature supply under dynamic demand in Experiments 19b and 19d (b). Supply of services exceeded demand throughout Experiment 19 except for regionalised supply of recreation under static demand.

**Figure 8:**
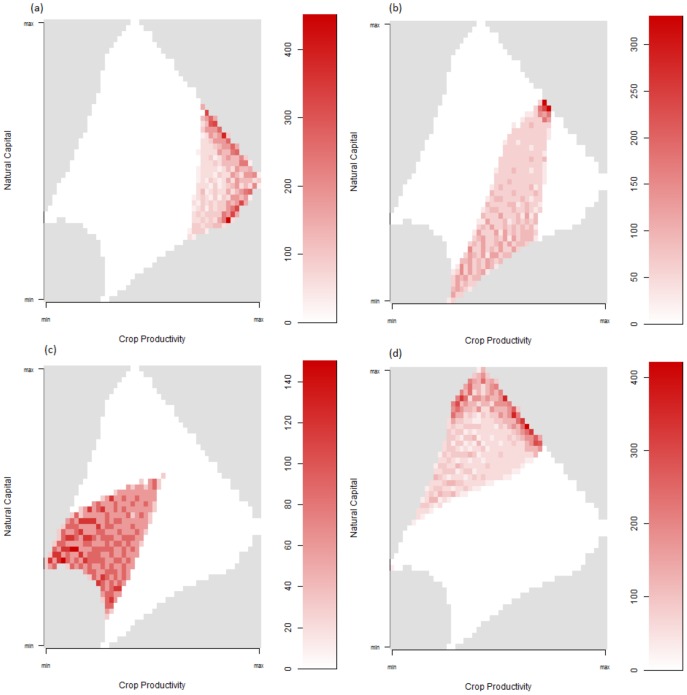
Agent locations in capital space in Experiment 19a. High-intensity farmers (a), mid-intensity farmers (b), low-intensity farmers (c) and conservationists (d), showing appropriate distributions relative to capital levels. Uniform grey areas do not occur in the modelled arena.

Raising the abandonment thresholds of high-intensity producers, without and with individual variation (Experiments 15 and 16 respectively), smoothed total productivities, reduced differences between global and regionalised productivities and slowed the pace of land use change (Fig. S4 in File S1; Table S1 in File S1). Regional production was maximised under uniform thresholds (and the consequent absence of high intensity producers that could not satisfy their minimum utility values). Varying the thresholds of the lower intensity producers (Experiments 17 and 18) introduced greater variation within and between realisations, and led to domination by either high or medium and low-intensity producers. The difference between regionalised and globalised systems was minimised when competition thresholds were increased (Fig. S5 in File S1). The final experiment (19) combined multi-functional agents with exponential utility functions. This dramatically increased ES production in all systems, with supply far exceeding demand in almost all cases (Figs. 7a & 7b). AFTs were distributed according to capital levels and productive ability (Fig. 8), and these distributions did not change substantially under dynamic demand. The configuration of land uses in the global case with static demands in this experiment represents a near-optimal outcome for the modelled system in terms of overall production levels.

## Discussion

The level and security of food supplies at national, regional and global levels are crucial issues, not only in their own right, but also because of their implications for economies, livelihoods, land use patterns and the production of other essential ecosystem services [34],[35]. In this study, we simulated contrasting production systems dedicated to maximising global food production or ensuring regional food security, and confronted these systems with sudden changes in demand levels and stylized models of human behaviour. These simulations allowed us to investigate some of the effects and trade-offs generated by each system in the presence of non-economically rational land manager behaviour and variations in land use intensity and multi-functionality, but in the absence of confounding effects that may occur in the real world.

The simulations presented here demonstrate that a completely globalised system of production can, in theory, supply more food from smaller areas of land than a system dedicated to ensuring regional food security. While regionalised systems did prevent the large-scale spatial separation of sites of supply and demand, they also generated overproduction in some regions and underproduction in others, with the relatively productive locations at risk of abandonment. Positive effects of global trade in food of the kind we find here are recognised [14], as are the negative effects of regionalisation or protectionism, which are known to include regional over-supply even under global shortages [11],[17], the abandonment of productive land [9], and the maintenance of unproductive or inefficient land uses [36]. Nevertheless, the benefits of regionalisation that we find in terms of spatial provision of food and other ecosystem services are also recognised, and indeed underpin many policies that support regional or national production [8].

Our findings also suggest that the above effects can depend strongly on the behaviour of individual land managers, and that the largest relative gains are to be made from globalisation when managers are homogeneous and rational in their response to demand for goods and services. This is known to be an inaccurate representation of land manager behaviour, and one that land use models are increasingly moving away from [37],[38]. We find that behavioural factors can, in principle, alter the level and security of food supply under globalised and regionalised systems in a number of different ways (Table 5). The majority of behaviours we considered decreased supply levels in the globalised case, but increased production per unit area of land, therefore improving the benefit individual land managers derived from their land and making more land available for other uses. In the case of variable intensities and multi-functional land uses, this was because managers could adapt their management to local conditions and potentially produce a wider range of services, as occurs in real land systems [39].

The strongest effect we found was that of raised abandonment thresholds (increased sensitivity to demand levels) under changing demand. Where we modelled a drop in demand affecting an AFT with a higher threshold, that type persisted only in the most productive areas, generating a highly concentrated pattern of land use and service provision. Where the demand drop affected an AFT with a lower threshold, however, agents became widely scattered, abandoning land in all areas and generating a fragmented pattern of land use with lower overall productive efficiency. Raised thresholds can describe any behaviour that makes land managers less willing or able to accept low returns on their activities (including the costs associated with the production of services or a change of land use). The dynamics we observed depend upon differences between types of land manager that are very likely to occur in reality; subsistence farmers, for example, have different priorities and costs, and so tolerate lower returns, than commercial farmers (e.g. [40]), while conservation is less sensitive to measurable returns than, say, forestry. We would therefore expect these groups to respond differently to changing demand levels, and this to result in different spatial configurations with strong implications for scale-dependent natural processes and service supply (e.g. [41],[42]). This could potentially also apply to similar land uses located in regions which differ in social characteristics that affect support for land managers, suggesting that policies concerned with food security should take account of their economic, behavioural and cultural context.

We also find that the disadvantages of strict regionalisation are diminished or even reversed under modelled forms of behaviour. Variations in competition thresholds (making agents less likely to relinquish their land to another agent) describe behaviour that is frequently observed in real land managers, and make productivity differences between globalised and regionalised cases smaller. These differences are decreased further by the use of exponential utility functions. Such functions, in guaranteeing positive utility for the overproduction of a good or service, can represent a number of factors: insensitivity to demand on the part of land managers (because of differing motivations for production, personal capital levels, support networks or the temporal scale of production, amongst others) [16],[43]; a failure of the trade system to transmit or express demand levels efficiently (e.g. [11]); or trade of surpluses beyond the boundaries of the modelled region or world (although there is an obvious limit to this ‘relaxed regionalisation’, below which this finding simply restates the theoretical advantages of globalisation). The number of factors that can contribute to this effect, and the likelihood of their occurrence, suggest that regional food security may not cause drops in overall production levels of the size estimated when fully rational economic behaviour is assumed.

Most dramatic, though, was the effect of introducing multi-functional land uses of varying intensity, which allowed simulated land managers to match their management to local conditions and improved production levels in the regional case. Multi-functionality is, of course, a characteristic of real-world land use, especially when ecosystem services are taken into account (e.g. [12],[44],[45]). Nevertheless, our findings suggest that its potential for increasing the supply and productivity of food and other ES may be large (beyond its obvious effects of increasing supply at local scales), and that it can represent a highly appropriate response to regionalisation in particular, allowing both production and security of supply to increase. It also appears to increase the sensitivity of the system to human behaviour and allow dramatic shifts in land use competitiveness, perhaps helping to explain the observed tendency of low-intensity producers to diversify when conditions are difficult [46],[47].

Our findings, of course, are not directly transferable from our simple simulated setting to the real world, and inferences about real-world processes must take account of the identities and forms of the factors included and excluded from the model (for instance our exclusion of general political or economic barriers to land use change). Most fundamentally, true globalisation of demand and supply is impossible, physically and politically (and strict regionalisation highly improbable), so that contrasting trade systems do not introduce or remove sensitivity of local land use to global factors, but instead vary the strength of this sensitivity [9]. Governments protect the interests of their own land managers and enact policies to preserve existing patterns of land use and ecosystem service supply, potentially constraining the ability of land managers to make ‘optimal’ decisions [7],[48]. Global food markets are, as a result, highly complex and relatively inert, comprising demands at many different spatial and temporal scales that produce ‘spaghetti bowls' of (limited) free trade between specific partners (e.g. [49],[50]). While some of our experiments might represent systems in which limited trade of surpluses occurs once regional supply levels are guaranteed, the complexities of more realistically structured systems could produce outcomes that differ dramatically from those we find [51]. The study of idealised theoretical systems at the extremes of the globalisation-regionalisation continuum allows us to understand basic characteristics of these systems that can inform interpretation of real-world phenomena, but such interpretation must be done with care.

In particular, our results are not intended to identify a form of land use system that is superior in any meaningful sense, and the theoretical advantages of globalised production systems that we identify are not necessarily sufficient to make the system desirable. The maximisation of productivity and efficiency does not necessarily ensure human or environmental wellbeing [52],[53]. Current land use trends are known to pose a serious threat to terrestrial and aquatic ecosystems [14],[44],[54] and globalisation can lead to insecurity in livelihoods and land use systems in the developed and developing world (e.g. [55],[56]), all of which disproportionately affect the poorest members of societies [57],[58]. Conversely, while regional food production can have benefits in terms of the security, stability and multi-functionality of land use systems (e.g. [8],[59]), its sensitivity to internal (e.g. behavioural) or external (e.g. climatic, political) factors and lower overall production levels may represent significant risks under the demographic, political and environmental changes currently affecting land systems [14].

In any case, globalisation is a rapid and continuing process, and globally-traded agricultural products increased in value from $32 billion in 1961 to $442 billion in 2002 [58]. Equally, human behaviour is known to be capable of confounding drivers of land use change and, more broadly, generating counter-intuitive systemic effects across apparently predictable systems [10],[19],[60],[61]. Our findings that various forms of land manager behaviour can dramatically alter the basic effects of complete globalisation and regionalisation of demand in a simulated setting are therefore highly relevant to current processes of land use change, particularly where the rate of change and implications for food supply, landscape heterogeneity, spatial provision of ecosystem services and the resilience of existing land uses are of concern.

## Conclusions

We find a number of strong effects of modelled land manager behaviour on stylised land use systems in globalised and regionalised settings. The most important of these include:

- Reductions in overall productivity, but increases in production per unit area, under globalisation, and increases in overall productivity under regionalisation, reducing the productivity gap between globalised and regionalised systems.
- Stabilisation of the land use system, with responses to changes in demand levels for ecosystem goods or services being reduced and/or slowed.
- A clear divergence in system-wide responses when sensitivity to demand levels varies between types of land managers, resulting either in concentration or fragmentation of similar land uses as demand levels change.
- The adaptation of land use intensity and multi-functionality to match local conditions, improving the spatial delivery of ecosystem services and overall productive efficiencies and totals.

The consequences of these effects for the production of food and other ecosystem goods and services are significant and, while our findings are only directly applicable to isolated behaviours in a simulated setting, clearly suggest that studies of land use change should take careful account of individual behaviour within the wider context.

## Supporting Information

File S1 **Supporting tables and figures as referred to in the text.**
(DOCX)
