# Supplementary material for: Experiments in Globalisation, Food Security and Land Use Decision Making
Source: PLoS One. 2014 Dec 1;9(12):e114213. doi: 10.1371/journal.pone.0114213 (PMC4250087; doi:10.1371/journal.pone.0114213)
Supplement: File S1 — Supporting tables and figures as referred to in the text. (DOCX) [file pone.0114213.s001.docx]

**Table S1**: Time taken (in terms of annual time steps) for production of each service to converge in each experiment under static and dynamic demand expressed globally and regionally (denoted as: GS = Global Static; GD = Global Dynamic; RS = Regional Static; RD = Regional Dynamic). The figures for dynamic demand show the time taken for convergence following a change in demand levels; initial convergence remains the same as in the equivalent static case. Convergence is defined as the point at which intra-annual variation between realisations exceeds inter-annual variation within them.

| **Experiment** | **GS time to convergence**  **Food; Recreation** | **GD time to convergence**  **Food; Recreation** | **RS time to convergence**  **Food; Recreation** | **RD time to convergence**  **Food; Recreation** |
| --- | --- | --- | --- | --- |
| 1 | 3; 3 | 1; 1 | 1; 1 | 0; 0 |
| 2 | 5; 1 | 0; 0 | 0; 0 | 0; 0 |
| 3 | 1; 5 | 0; 1 | 0; 0 | 0; 0 |
| 4 | 4; 4 | 0; 0 | 0; 0 | 0; 0 |
| 5 | 1; 1 | 1; 1 | 0; 0 | 0; 0 |
| 6 | 3; 3 | 1; 1 | 1; 1 | 0; 0 |
| 7 | 1; 1 | 1; 1 | 1; 1 | 0; 0 |
| 8 | 0; 0 | 2; 2 | 0; 0 | 0; 0 |
| 9 | 5; 5 | 1; 1 | 0; 0 | 0; 0 |
| 10 | 0; 4 | 0; 1 | 0; 0 | 0; 0 |
| 11 | 2; 3 | 1; 1 | 0; 0 | 0; 1 |
| 12 | 3; 3 | 3; 3 | 0; 0 | 0; 0 |
| 13 | 3; 3 | 3; 3 | 0; 0 | 0; 0 |
| 14 | 9; 11 | 0; 3 | 0; 0 | 0; 0 |
| 15 | 2; 5 | 10; 9 | 0; 0 | 0; 0 |
| 16 | 3; 4 | 9; 11 | 0; 0 | 0; 0 |
| 17 | 1; 2 | 0; 0 | 0; 0 | 0; 0 |
| 18 | 3; 3 | 1; 8 | 0; 0 | 0; 0 |
| 19 | 0; 2 | 1; 1 | 0; 0 | 0; 0 |

**Figure S1**: Supply of food under dynamic recreation demand levels in Experiment 3 (a), where conservationists had a uniform giving-up threshold, and in Experiment 5 (b), where conservationists had a variable giving-up threshold centered on its value in Experiment 3.


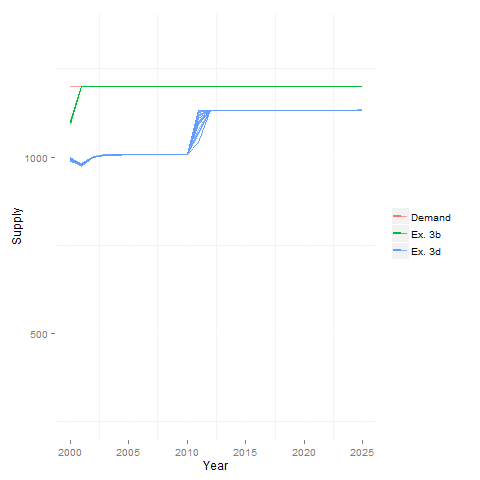

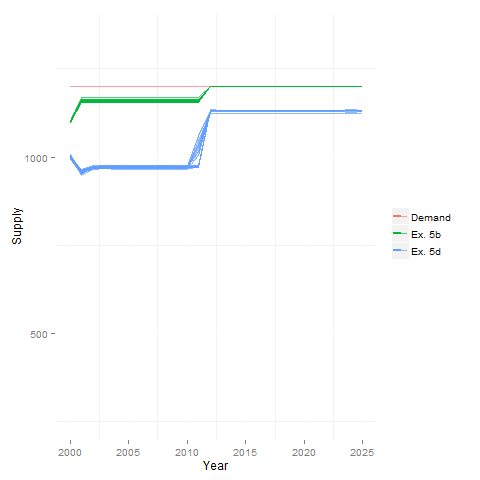


(a)

(b)

**Figure S2**: Supply of food under dynamic recreation demand levels in Experiment 7 (a), showing gradual increase in supply prior to demand level change, and the final distribution of farmer agents in capital space in Experiment 7b (b). Grey areas of capital space in (b) do not occur in the modelled arena.


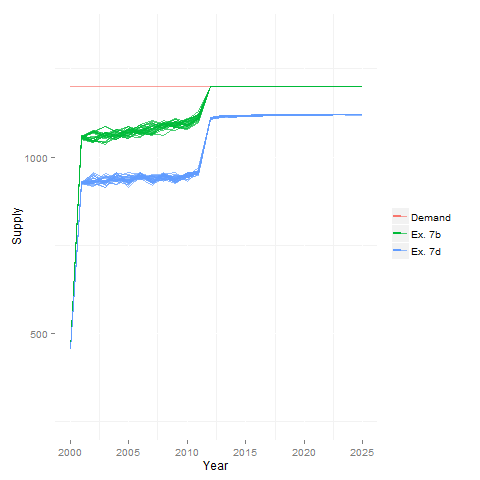


(b)

(a)


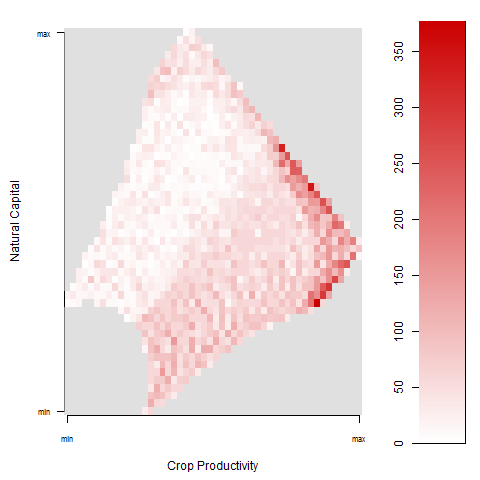


**Figure S3**: Examples of final agent maps in Experiment 14 under global static demand (14a; (a)) and regional dynamic demand (14(d); (b)), and the supply of food under global static demand (14(a); (c)) and global dynamic demand (14b; (d)).


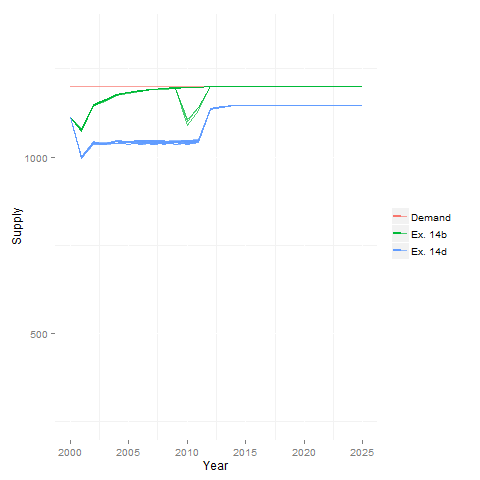

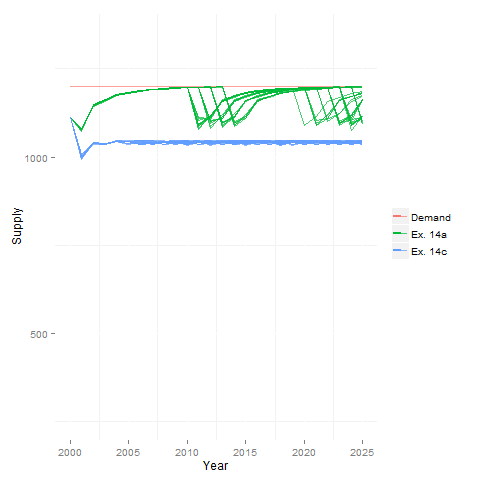

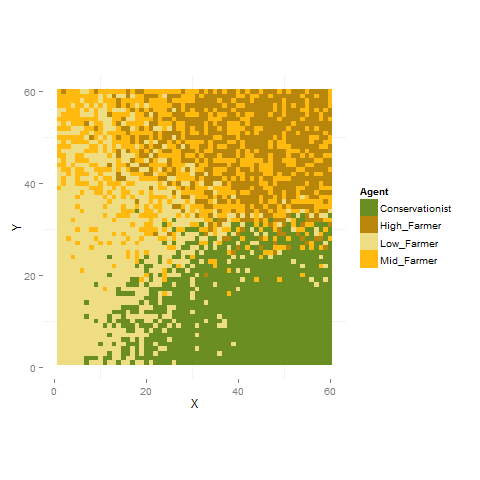

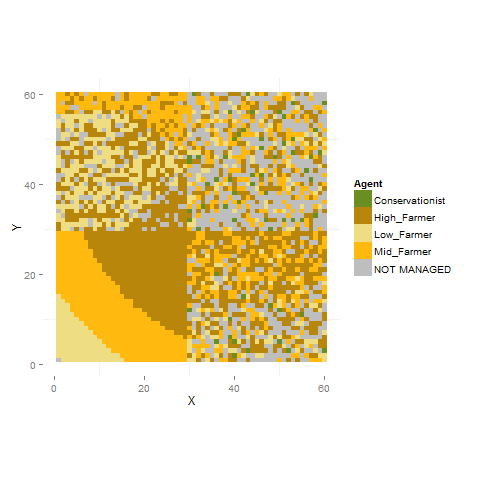


(a)

(b)

(c)

(d)

**Figure S4**: Service supply under dynamic demand in Experiments 15b and 15d, for food (a) and recreation (b). Demand is in red.


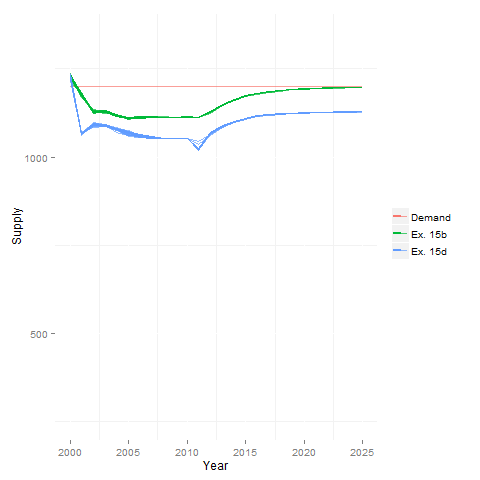


(a)

(b)


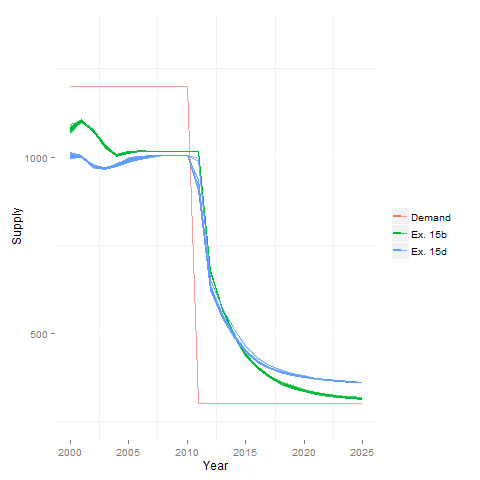


**Figure S5**: Food supply under static demand in Experiments 17 (a) and 18 (b).


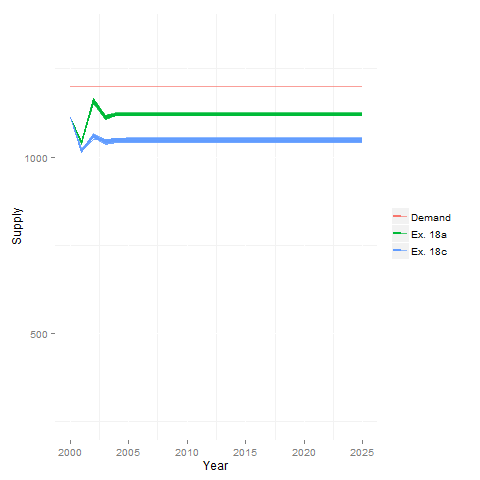

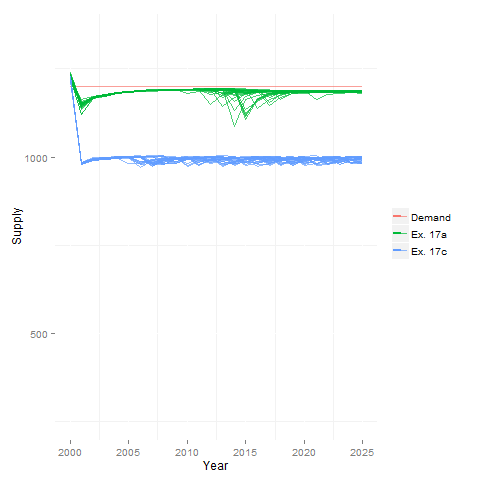


(a)

(b)
